# Drinking Ice Water

**Published:** 1859-09

**Authors:** 


### DRINKING ICE WATER.

A gentleman from Minnesota assures us that for four five years, he and all his family have drank most freely of ice water all the year round, without any noticeable inconvenience, enjoying the meanwhile, excellent health.

We advise all persons to avoid drinking ice water at all times, because—First, many have fallen dead from drinking pump or spring water freely while in a heat, and we chose in the pages of the *Journal of Health*, to be on the safe side always, if possible. Second: It is known by ocular proof on the part of scientific men, that the process of digestion in the stomach is arrested on the very instant of cold water being taken into it, and that that process is not resumed, until enough heat has been taken from the general system, to raise the water from thirty-two or more degrees, up to one hundred. In the case of feeble persons, this would require an hour or more; where there was not power enough for re-action, the person has arisen from the table in a chill, ending fatally.

Within a few weeks, General Bruat, while leading a part of the French army into Italy, all heated with the effort of climbing the mountain, drank down a glass of snow water, and fell as if he had been stricken by a shot, dying at the instant.

If any of this ice water family are living in sound health fifty years hence, with their present drinking habits continued in the meanwhile, it may be a valuable practical fact. Some people have survived bullets, rail car collisions, and the blowing up of steamboats. No one rule is suitable for all. We aim to give advice applicable to the great majority of the people, advice founded on observations extended, and repeated, and testified to by competent and reliable men. We hope never to give advice on single cases, or possibilities, or conjectures, or "may be's." Nor will we knowingly put any of our readers on an experimental course. We will aim hereafter as heretofore, to deal only in the known, the corroborated and the safe.

